# Induction of creatine kinase release from cultured osteoclasts via the pharmacological action of aminobisphosphonates

**DOI:** 10.1186/s40064-015-0848-3

**Published:** 2015-02-03

**Authors:** Makoto Tanaka, Hiroshi Mori, Ryoji Kayasuga, Kazuhito Kawabata

**Affiliations:** Research Promotion, Ono Pharmaceutical Co., Ltd, 3-1-1, Sakurai, Shimamoto-cho, Mishima-gun Osaka, 618-8585 Japan; Discovery Research Laboratories, Ono Pharmaceutical Co., Ltd, Shimamoto-cho, Mishima-gun Osaka, 618-8585 Japan

**Keywords:** Osteoclast, Bisphosphonate, Creatine kinase, Minodronic acid, Alendronate, Risedronate

## Abstract

An increase of serum creatine kinase (CK) has been observed in clinical studies of nitrogen-containing aminobisphosphonates (N-BPs). Osteoclasts are thought to be the source of the CK, but there is no clear evidence for the hypothesis. In this study, CK release from rabbit osteoclasts induced by N-BPs was examined in an *in vitro* culture system. Rabbit bone-derived cells were cultured for 3 days on the N-BPs pretreated cortical bone slices. CK activity in the culture medium was measured at 3 days of culture. The CK activity was increased with all N-BPs at concentrations at which showed antiresorptive activity over 60% inhibition of C-terminal cross-linking telopeptide of type I collagen (CTX-1) release. The maximum induction of CK activity was 2.6 times the control level. The lowest N-BP concentration inducing CK release was 3 times lower than that required to decrease the osteoclast number. Bafilomycin A1, an inhibitor of vacuolar H^+^-ATPase, abrogated all N-BP actions, including CK release. Bone-derived cells except osteoclasts were insensitive to bafilomycin A1, suggesting that osteoclasts were the source of CK. Regarding the time course, CK release occurred after a 1 day lag time and increased steadily until day 3 of culture. These results show that CK release is induced by N-BPs from osteoclasts at concentrations at which N-BPs show antiresorptive activity over 60% inhibition of CTX-1 release *in vitro*. These findings explain the mechanism of the CK increase induced by clinical use of N-BPs.

**Electronic supplementary material:**

The online version of this article (doi:10.1186/s40064-015-0848-3) contains supplementary material, which is available to authorized users.

## Introduction

Creatine kinase (CK) is a dimeric enzyme of about 86 kDa that catalyzes the reaction of creatine and adenosine triphosphate (ATP) to form phosphocreatine and adenosine diphosphate (ADP), a crucial reaction for cellular energy generation and metabolism (Wallimann et al. 1992). The enzyme has three isoenzymes: ubiquitous brain-type creatine kinase (CK-BB); sarcomeric muscle type (CK-MM) and cardiac muscle type (CK-MB) (Eppenberger et al. 1967).

Mature osteoclasts are multinucleated, giant cells that resorb bone based on synthesis and secretion of acid and degradative enzymes (Teitelbaum 2007), and maintain a high energy state for active bone resorption (Hazama et al. 2009). The CK-BB gene is highly expressed in rabbit osteoclasts (Sakai et al. 1995) and CK-BB is the predominant during receptor activator of NF-κB ligand (RANKL)-induced osteoclastogenesis in mouse (Chen et al. 2010). In immunohistochemical analysis, CK-BB expression in osteoclasts has been shown in bone sections (Sistermans et al. 1995). Moreover, decreasing CK-BB gene expression by RNA interference or blocking of activity by cyclocreatine, an inhibitor of CK-BB, suppresses bone resorption by osteoclasts grown *in vitro* (Chang et al. 2008). The blocking was considered via effects on actin ring formation, RhoA GTPase activity, and vacuolar H^+^-ATPase function. Furthermore, CK-BB is present in serum in patients with osteopetrosis and in some fetal and malignant tissues, and abnormal osteoclasts may be a potential source of circulating CK-BB in osteopetrosis (Yoneyama et al. 1989; Whyte et al. 1996). Increases in tartrate-resistant acid phosphatase (TRAP) and CK-BB in serum were occurred in osteopetrosis (Waguespack et al. 2002).

Aminobisphosphonates (N-BPs) are widely used drugs for treatment of osteoporosis because of their strong inhibition of accelerated bone resorption and the consequent increase in bone mineral density and prevention of fracture (Black et al. 1996; Reginster et al. 2000; Matsumoto et al. 2009). Two phosphate side chains on the central carbon atom in the P–C–P backbone are mainly responsible for binding to bone, with complementary interactions through a hydroxyl group (Rogers 2003; Russell et al. 2008). N-BPs also have a nitrogen-containing side chain on the central carbon that determines the inhibitory potency for a target enzyme in the mevalonate pathway, farnesyl pyrophosphate (FPP) synthase. Therefore, the side chains determine the antiresorptive potency (Ebetino et al. 2011). Bone-bound N-BPs are internalized by bone-resorbing osteoclasts and reduce the bone resorption activity or viability of the cells through inhibition of FPP synthase. Release of bone-bound N-BPs is promoted by vacuolar H^+^-ATPase, which is localized along the ruffled borders of osteoclasts and pumps protons out onto the bone surface, and this release can be inhibited by inhibitors of V-ATPase such as bafilomycin A1 (Takami et al. 2003).

An increase in the serum CK level has been observed in clinical studies of N-BPs. The frequency of CK elevation was between 1% and less 5% in a trial of 5 mg alendronate (in Japanese package insert), while increases of CK-BB and CK-MB were found in single and multiple oral dose phase I studies of risedronate in Japanese healthy adult male subjects (Ogura et al. 2004). Minodronic acid treatment at 1 mg has been found to elevate CK at a frequency of less 1% (in Japanese package insert). Thus, many reports have shown CK elevation during N-BP treatment, but the mechanism is unclear. We hypothesized that CK is released from osteoclasts, but not from other cells present in bone. To test this hypothesis, we examined CK release induced by N-BPs from rabbit bone-derived cells *in vitro*, and we assessed the effect of bafilomycin A1 on CK release to determine the source of CK. We also examined the time course of CK release after treatment with N-BPs.

## Materials and methods

### Reagents

Alendronate and risedronate were purchased from LKT laboratories (St. Paul, MN, USA). Minodronic acid was supplied by Astellas Pharma (Tokyo, Japan). Bafilomycin A1 was obtained from Wako Pure Chemical Industries (Osaka, Japan). Reagents for TRAP staining were purchased from Sigma Chemical (St. Louis, MO, USA). Alpha-modified Eagle's medium (α-MEM) and fetal bovine serum (FBS) were purchased from Gibco (Grand Island, NY, USA).

### Preparation of osteoclast-containing bone cells

Unfractionated bone cells were isolated from femurs, tibias, humeri, ulnas, and radii of 10-day-old Kbl:NZW rabbits (Kitayama Labes, Nagano, Japan) using a reported method (Kakudo et al. 1996) with minor modifications. Briefly, the long bones of rabbits were minced for 10 min in α-MEM plus 5% FBS as culture medium, after soft connective tissues were removed. Bone cells were dissociated from bone fragments by vigorous vortexing for 1 min. After sedimentation of the bone fragments for 2 min under normal gravity, a supernatant containing the released cells was obtained. Bone cells were collected by centrifugation twice at 40 *g* for 2 min. The cells were prepared as 5 × 10^6^ viable cells/mL. Animal studies were conducted in compliance with the Guidelines for Animal Studies established by Research Headquarters, Ono Pharmaceutical Co., Ltd (Osaka, Japan).

### Cortical bovine bone slices

Cortical bone slices were cut from bone sticks prepared from bovine femoral cortical bone. The sticks were cut into slices of thickness 0.15 mm using an Isomet low speed saw (Buehler, Lake Bluff, IL, USA). The bone slices had a 6-mm diameter for fitting into 96-well plates. The slices were sterilized in 70% ethanol and pre-incubated in culture medium. The surface area of each cortical bone slice was calculated as 59.3 mm^2^.

### Treatment of cortical bone slices with N-BPs

Bone slices were treated for 19 h with 150 μL culture medium containing alendronate (3–30 μmol/L), risedronate (1–10 μmol/L), or minodronic acid (0.3–3 μmol/L).

### Pit formation assay

Culture medium (100 μL) containing bone cells (5 × 10^5^) was added onto cortical bone slices pre-treated with N-BPs. These slices had previously been soaked in 50 μL of culture medium in a 96-well plate. After incubation in a CO_2_ incubator (5% CO_2_, 95% air) at 37°C for 2 h, the 150 μl of culture medium was replaced with fresh culture medium and incubated for 3 more days. In experiments using bafilomycin A1, the culture medium was refreshed with or without bafilomycin A1 (10 nmol/L). In the time course study, the culture medium was changed to fresh medium every day, except in the whole treatment (0–3 days) group.

### Creatine kinase assay

CK activity was measured with a CK Test Wako (Wako Pure Chemical Industries Ltd., Osaka, Japan). Each CK activity was calculated by subtracting the background in culture medium and bone slices. One unit was defined as the amount of enzyme that catalyzed reaction of 1 μmol/L substrate in 1 min at 37°C.

### Measurement of bone resorption

Bone resorption was measured based on release of C-terminal cross-linking telopeptide of type I collagen (CTX-1) during culture. The CTX-I level in culture supernatant was determined using a CrossLaps for Culture kit (Nordic Bioscience Diagnostics A/S, Herlev, Denmark) with samples in duplicate wells. The CTX-1 level was calculated by subtracting the background in culture medium, bone slices and bone-derived cells.

### Measurement of osteoclast number

TRAP staining was used for osteoclast quantification. Briefly, osteoclasts were fixed for 1 h by soaking bone slices in 4% paraformaldehyde solution. After washing with phosphate buffered saline (PBS (-)) and PBS (-) with 0.1% Triton X-100, osteoclasts were stained by incubation with naphthol AS-BI phosphate in dimethylformamide in acetate-tartrate buffer for 10 min at 37°C. Osteoclasts numbers were quantified as the number of TRAP-positive multinuclear cells on bone slices. TRAP-stained osteoclasts in bone slices were counted using an upright microscope (Olympus, Tokyo, Japan) under blinded conditions.

### Statistics

All data are presented as means ± SE. Statistical analyses were performed using SAS System Release 6.10 or above. In the bafilomycin A1 untreated experiment, differences between control and treated group in each N-BP were analyzed by Williams test. In the bafilomycin A1 treated experiment, differences between groups, minodronic acid treated groups and control group without or with bafilomycin A1, were analyzed by Tukey test, except for use of a Steel-Dwass test for osteoclast number. Differences were considered significant at *p* < 0.05.

## Results

### Effects of N-BPs on bone resorption and CK release in a rabbit pit assay

The CTX-1 level, an index of bone resorption activity, and CK release were measured in an *in vitro* rabbit bone-derived cell culture on cortical bone slices pretreated with three N-BPs. The concentrations of alendronate (3–30 μmol/L), risedronate (1–10 μmol/L) and minodronic acid (0.3–3 μmol/L) were selected based on study showing roughly 50% CTX-1 inhibition at the lowest N-BP concentrations (Halasy-Nagy et al. 2001; Nozaki et al. 2008). Each N-BP dose-dependently decreased the CTX-1 level (Table 1). Over 50% inhibition of CTX-1 level was achieved by 10 μmol/L of alendronate, ≤1 μmol/L of risedronate and ≤0.3 μmol/L of minodronic acid. Inhibitory ratios of minodronic acid on CTX-1 level were higher than that of risedronate at both 1 and 3 μmol/L. Consequently, the most potent antiresorptive N-BPs in the test was minodronic acid, followed in order by risedronate and alendronate. Each N-BP also dose-dependently increased CK release. A maximum CK release, rose up to 2.6 times the control level, was occurred at the highest concentration of minodronic acid. Significant CK release was occurred all tested N-BPs at concentrations giving over 60% inhibition of CTX-1 level.

**Table 1:** **Effects of aminobisphosphonates on bone resorption and CK activities in rabbit bone cells culture**

**Treatment**	**Concentration of N-BP (μmol/L)**	**CTX-1**		**CK activity (U/L)**
		**Concentration (nmol/L)**	**% Inhibition**	
Control	-	201.1 ± 8.5	-	9.7 ± 0.3
Alendronate	3	135.1 ± 6.5	32.8	10.0 ± 0.2
	10	58.1 ± 6.0	71.1	12.2 ± 0.4^#^
	30	25.8 ± 1.4	87.2	13.3 ± 0.6^#^
Risedronate	1	89.4 ± 5.9	55.5	10.8 ± 0.4
	3	37.5 ± 3.8	81.4	12.6 ± 0.2^#^
	10	17.4 ± 2.2	91.3	16.4 ± 0.6^#^
Minodronic acid	0.3	61.8 ± 4.0	69.3	15.9 ± 0.8^#^
	1	25.8 ± 4.6	87.2	22.5 ± 1.4^#^
	3	6.5 ± 1.0	96.8	25.0 ± 0.7^#^

Values represents the mean ± SE of 8 individual cultures.

^#^Significantly different from control group (p < 0.05).

### Effects of bafilomycin A1 with minodronic acid in a rabbit pit assay

**Figure 1:**
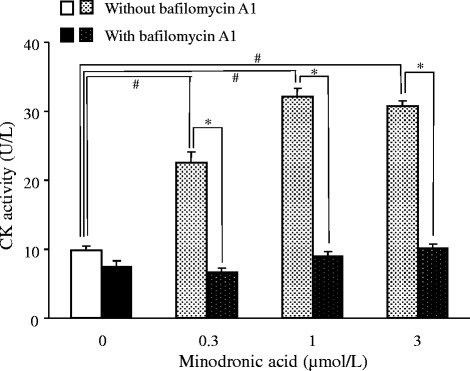
**Effects of minodronic acid and bafilomycin A1 on CK activity in rabbit bone-derived cells.** Each column represents the mean ± SE of 10 individual cultures. #:Significantly different between control and minodronic acid treated groups in bafilomycin A1 untreated condition (*p* < 0.05). *:Significantly different between corresponding bafilomycin A1 treated and untreated groups (*p* < 0.05).

**Figure 2:**
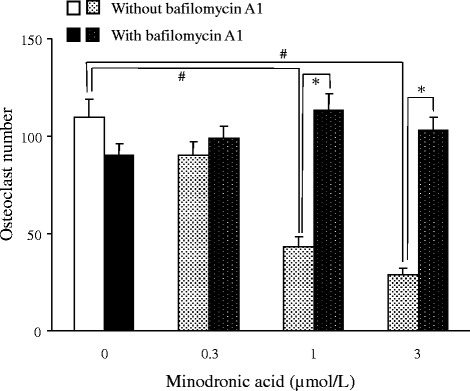
**Effects of minodronic acid and bafilomycin A1 on osteoclast number in rabbit bone-derived cells.** Each column represents the mean ± SE of 10 individual cultures. #:Significantly different between control and minodronic acid treated groups in bafilomycin A1 untreated condition (*p* < 0.05). *:Significantly different between corresponding bafilomycin A1 treated and untreated groups (*p* < 0.05).

The effect of bafilomycin A1 on the CTX-1 level, CK activity and osteoclast number in cultured bone-derived cells were examined to clarify the source of CK release as osteoclasts. In the absence of bafilomycin A1, minodronic acid dose-dependently decreased the CTX-1 level (Table 2). The CTX-1 release was completely abolished by addition of bafilomycin A1 into the culture. Minodronic acid dose-dependently increased the CK activity, and the increased CK activity was also cancelled by bafilomycin A1 addition (Figure 1). Furthermore, minodronic acid reduced TRAP positive osteoclast number at 1 μmol/L or more (Figure 2). The decrease in osteoclast number by minodronic acid was completely abolished by addition of bafilomycin A1 into the culture.

**Table 2:** **Effects of bafilomycin A1 on bone resorption in rabbit bone cells culture**

**Treatment**	**Concentration of minodronic acid (μmol/L)**	**CTX-1 concentration (nmol/L)**	
		**Without bafilomycin A1**	**With bafilomycin A1**
Control	-	265.0 ± 19.2	3.0 ± 0.6*
Minodronic acid	0.3	50.9 ± 4.3^#^	3.2 ± 0.4*
	1	14.0 ± 1.4^#^	2.4 ± 0.5*
	3	6.2 ± 1.1^#^	2.5 ± 0.3*

Values represents the mean ± SE of 10 individual cultures.

^#^Significantly different from bafilomycin A1 untreated control (*p* < 0.05).

*Significantly different from corresponding bafilomycin A1 untreated group (*p* < 0.05).

### Time course of CK release induced by minodronic acid in a rabbit pit assay

**Figure 3:**
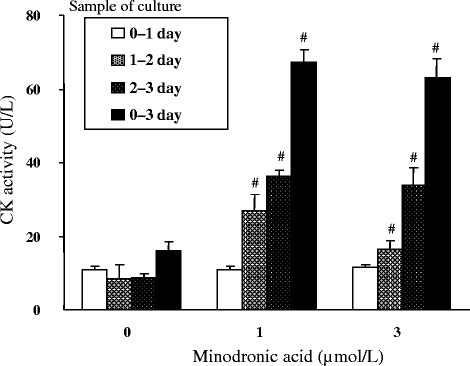
**Time course study of CK release from rabbit bone-derived cells after treatment with minodronic acid.** Each column represents the mean ± SE of 6 individual culture of indicated incubation periods. #:Significantly different between control and minodronic acid treated groups within corresponding incubation period (*p* < 0.05).

The time course of CK release was analyzed in bone-derived cell culture with minodronic acid treatment. CK activities were measured in culture mediums obtained on days 0–1, 1–2, 1–3, and 0–3 (Figure 3). CK activity was not increased during day 0–1, but was increased minodronic acid treatment during days 1–2 and 2–3.

## Discussion

The most potent antiresorptive N-BPs in the test was minodronic acid, followed in order by risedronate and alendronate. These findings are similar to the order of potency for inhibition of FPP synthase (Dunford et al. 2001; Ohno et al. 2011) and effects in rabbit osteoclast cultures (Halasy-Nagy et al. 2001; Nozaki et al. 2008). Contrary to N-BPs have different hydroxyapatite binding affinities (Ebetino et al. 2011), antiresorptive potency on bone slices was same orders to the FPP synthase inhibition. This suggests that the influence on binding affinities to hydroxyapatite may be smaller than difference of antiresorptive activities. Despite there is difference on antiresorptive potency in N-BPs, the minimum concentration for induction of CK release was approximately that required over 60% inhibition of CTX-1 release for all three N-BPs.

Ito et al. found that CK release was induced by 3 mmol/L incadronate, but not by alendronate, in a rabbit osteoclast culture on a plastic plate (Ito et al. 1998). In contrast, CK was released in culture with 10–30 μmol/L of alendronate in this study. We and others have shown antiresorptive activity at 3–30 μmol/L alendronate in rabbit osteoclasts cultured on bovine cortical bone slices (Halasy-Nagy et al. 2001). The concentration of alendronate required for antiresorptive action and CK release on bone slices was 1000 times lower than that used by Ito et al., the culture condition of the osteoclasts may cause significant difference of results. The decrease in osteoclast number occurred at 1 μmol/L, while the IC_50_ of minodronic acid for CTX-1 release in a rabbit pit assay is 0.11 μmol/L (Nozaki et al. 2008); therefore, the concentration of minodronic acid required to decrease the osteoclast number was roughly 10 times higher than the IC_50_ for CTX-1 release. The alendronate and risedronate concentrations required to decrease the osteoclast number were also roughly 10 times higher than the respective IC_50_ values for CTX-1 release (Halasy-Nagy et al. 2001). These results suggest that the dose ratios for antiresorptive action and decrease of osteoclast number are similar for all N-BPs.

The intracellular pathway for the antiresorptive effect of N-BPs occurs via prenylation of small G proteins like Rab (Nishida et al. 2003; Coxon et al. 2001), while that for apoptosis involves extracellular signal-regulated kinase (ERK) and Bim, Bcl-2 homology 3 (BH3)-only protein (the ERK/Bim axis) (Matsumoto et al. 2011). The CK release is related to cellular damage such as that due to apoptosis, but the N-BPs concentration for CK release was 0.3 μmol/L for minodronic acid, which is 3 times lower than that required for a decrease of osteoclast number. However, histological changes related to osteoclasts apoptosis was observed at 0.1 μmol/L of minodronic acid (Additional file 1). Since the decrease in osteoclast number is considered to be a consequence of osteoclast apoptosis (Ito et al. 1999), the parameter may be less sensitive for cellular damage and thus a high concentration of N-BPs is needed to decrease the osteoclast number. CK release from osteoclasts also had a 1-day lag time and occurred during days 1 to 3 of culture, with a maximum from days 2 to days 3. Morphological changes of osteoclasts are sequential, with initial actin ring disruption related to an antiresorptive effect within 1 day after treatment, and subsequent nuclear condensation after an approximately 1-day lag time, with maximum apoptosis on day 3 (Halasy-Nagy et al. 2001; Tsubaki et al. 2013). Hence, all examined N-BPs induced CK release at micromolar to tens of micromolar concentrations in association with osteoclast apoptosis and bone resorption. The 1-day lag time and maximum response on day 3 (as N-BPs induced apoptosis) indicates that CK release is an osteoclast apoptosis-related event.

Mature osteoclasts create an isolated microenvironment between themselves and the bone surface, into which the cells secrete protons via vacuolar H^+^-ATPase and the lysosomal protease cathepsin K. Bone-bound N-BPs are eluted by acid during bone resorption and may be incorporated into osteoclasts, and osteoclasts-incorporate N-BPs show antiresorptive activity and cause apoptosis. All pharmacological action of minodronic acid, inhibition of CTX-1 release, reduction of osteoclast number and CK release were abrogated by the V-ATPase inhibitor bafilomycin A1. The antiresorptive action and CK release induced by alendronate and risedronate were also eliminated by bafilomycin A1 (data not shown). Because acid release from osteoclasts was inhibited by bafilomycin A1, N-BPs were not released from bone slices and not incorporated into osteoclasts in bafilomycin A1 treated condition. Besides osteoclasts, stromal cells and bone marrow cells were present in the culture system, but these cells are not sensitive to bafilomycin A1. Bafilomycin A1 also blocked vesicle trafficking with sufficient inhibition at approximately 50 nmol/L or more, but the effect was minimal at our used condition around 10 nmol/L (Johnson et al. 1993). In addition, Sistermans et al. reported no CK-BB was found in both osteocyte and cartilage, and general CK-BB expression in bone was negative (Sistermans et al. 1995). Therefore, these results suggest that CK is released from osteoclasts via the pharmacological effect of N-BPs. We did not identify CK isoenzymes because of the limited CK activity, and this is a limitation of the study. However, expression of CK except CK-BB was tissue specific. The ubiquitous CK-BB is strongly expressed in osteoclasts and it is likely that CK-BB was released from damaged osteoclasts into extracellular fluid due to N-BP treatment. The serum CK level increases in some patients treated with N-BPs and serum CK-BB might be a useful marker for osteoclast damage in N-BP treatment.

Regarding the CK-BB and ATP axis, the importance of ATP function in osteoclasts and osteoblasts is becoming clearer. Intracellular ATP levels play a pivotal role in osteoclast apoptosis and in the bone-resorbing function of osteoclasts (Miyazaki et al. 2012). In phagocytes, local cytoskeletal dynamics during cell motility is coupled to onsite availability of ATP generated by CK-BB (Kuiper et al. 2008). Therefore, CK-BB and ATP are important in osteoclast bone resorption. N-BP-induced damage in osteoclasts may cause extracellular release of a high concentration of ATP. Osteoclasts and osteoblasts express various members of the P2 receptor family, for which ATP is a physiological ligand (Burnstock et al. 2013). Moreover, P2 receptors are involved in osteoclast fusion (Pellegatti et al. 2011) and P2x receptors may negatively regulate bone mineralization in osteoblasts (Orriss et al. 2012). Thus, a high concentration of ATP from damaged osteoclasts might underlie N-BPs induced giant osteoclast formation (Weinstein et al. 2009), and might influence on cross-talk between osteoblasts and osteoclasts.

## Conclusion

N-BPs induced CK release at an antiresorptive activity over 60% inhibition of CTX-1 in *in vitro* culture. The CK release was considered to be derived from osteoclasts, because it had a 1 day lag time and was blocked by bafilomycin A1. These findings support the possible mechanism of CK elevation by N-BPs in the clinic.

## Additional file

Additional file 1:
**Induction of Creatine Kinase Release from Cultured Osteoclasts via the Pharmacological Action of Aminobisphosphonates.**

## References

[CR1] Black DM, Cummings SR, Karpf DB, Cauley JA, Thompson DE, Nevitt MC, Bauer DC, Genant HK, Haskell WL, Marcus R, Ott SM, Torner JC, Quandt SA, Reiss TF, Ensrud KE (1996). Randomised trial of effect of alendronate on risk of fracture in women with existing vertebral fractures. Fracture Intervention Trial Research Group. Lancet.

[CR2] Burnstock G, Arnett TR, Orriss IR (2013). Purinergic signalling in the musculoskeletal system. Purinergic Signal.

[CR3] Chang EJ, Ha J, Oerlemans F, Lee YJ, Lee SW, Ryu J, Kim HJ, Lee Y, Kim HM, Choi JY, Kim JY, Shin CS, Pak YK, Tanaka S, Wieringa B, Lee ZH, Kim HH (2008). Brain-type creatine kinase has a crucial role in osteoclast-mediated bone resorption. Nat Med.

[CR4] Chen J, Sun Y, Mao X, Liu Q, Wu H, Chen Y (2010). RANKL up-regulates brain-type creatine kinase via poly(ADP-ribose) polymerase-1 during osteoclastogenesis. J Biol Chem.

[CR5] Coxon FP, Helfrich MH, Larijani B, Muzylak M, Dunford JE, Marshall D, McKinnon AD, Nesbitt SA, Horton MA, Seabra MC, Ebetino FH, Rogers MJ (2001). Identification of a novel phosphonocarboxylate inhibitor of Rab geranylgeranyl transferase that specifically prevents Rab prenylation in osteoclasts and macrophages. J Biol Chem.

[CR6] Dunford JE, Thompson K, Coxon FP, Luckman SP, Hahn FM, Poulter CD, Ebetino FH, Rogers MJ (2001). Structure-activity relationships for inhibition of farnesyl diphosphate synthase in vitro and inhibition of bone resorption in vivo by nitrogen-containing bisphosphonates. J Pharmacol Exp Ther..

[CR7] Ebetino FH, Hogan AM, Sun S, Tsoumpra MK, Duan X, Triffitt JT, Kwaasi AA, Dunford JE, Barnett BL, Oppermann U, Lundy MW, Boyde A, Kashemirov BA, McKenna CE, Russell RG (2011). The re lationship between the chemistry and biological activity of the bisphosphonates. Bone.

[CR8] Eppenberger HM, Dawson DM, Kaplan NO (1967). The comparative enzymology of creatine kinases. I. Isolation and characterization from chicken and rabbit tissues. J Biol Chem..

[CR9] Halasy-Nagy JM, Rodan GA, Reszka AA (2001). Inhibition of bone resorption by alendronate and risedronate does not require osteoclast apoptosis. Bone.

[CR10] Hazama R, Qu X, Yokoyama K, Tanaka C, Kinoshita E, He J, Takahashi S, Tohyama K, Yamamura H, Tohyama Y (2009). ATP-induced osteoclast function: the formation of sealing-zone like structure and the secretion of lytic granules via microtubule-deacetylation under the control of Syk. Genes Cells.

[CR11] Ito M, Chokki M, Ogino Y, Satomi Y, Azuma Y, Ohta T, Kiyoki M (1998). Comparison of cytotoxic effects of bisphosphonates in *vitro* and *in vivo*. Calcif Tissue Int.

[CR12] Ito M, Amizuka N, Nakajima T, Ozawa H (1999). Ultrastructural and cytochemical studies on cell death of osteoclasts induced by bisphosphonate treatment. Bone.

[CR13] Johnson LS, Dunn KW, Pytowski B, McGraw TE (1993). Endosome acidification and receptor trafficking: bafilomycin A1 slows receptor externalization by a mechanism involving the receptor's internalization motif. Mol Biol Cell.

[CR14] Kakudo S, Miyazawa K, Kameda T, Mano H, Mori Y, Yuasa T, Nakamura Y, Shiokawa M, Nagahira K, Tokunaga S, Hakeda Y, Kumegawa M (1996). Isolation of highly enriched rabbit osteoclasts from collagen gels: A new assay system for bone-resorbing activity of mature osteoclasts. J Bone Miner Metab.

[CR15] Kuiper JW, Pluk H, Oerlemans F, van Leeuwen FN, de Lange F, Fransen J, Wieringa B (2008). Creatine kinase-mediated ATP supply fuels actin-based events in phagocytosis. PLoS Biol.

[CR16] Matsumoto T, Hagino H, Shiraki M, Fukunaga M, Nakano T, Takaoka K, Morii H, Ohashi Y, Nakamura T (2009). Effect of daily oral minodronate on vertebral fractures in Japanese postmenopausal women with established osteoporosis: a randomized placebo-controlled double-blind study. Osteoporos Int.

[CR17] Matsumoto T, Nagase Y, Iwasawa M, Yasui T, Masuda H, Kadono Y, Nakamura K, Tanaka S (2011). Distinguishing the proapoptotic and antiresorptive functions of risedronate in murine osteoclasts: role of the Akt pathway and the ERK/Bim axis. Arthritis Rheum.

[CR18] Miyazaki T, Iwasawa M, Nakashima T, Mori S, Shigemoto K, Nakamura H, Katagiri H, Takayanagi H, Tanaka S (2012). Intracellular and extracellular ATP coordinately regulate the inverse correlation between osteoclast survival and bone resorption. J Biol Chem.

[CR19] Nishida S, Kikuichi S, Haga H, Yoshioka S, Tsubaki M, Fujii K, Irimajiri K (2003). Apoptosis-inducing effect of a new bisphosphonate, YM529, on various hematopoietic tumor cell lines. Biol Pharm Bull.

[CR20] Nozaki K, Mori M, Chono K, Tanaka S, Fukushima S, Sasamata M, Kayasuga R, Mori H, Tanaka M, Kawabata K (2008). Mechanism of pharmacologic action of minodronic acid. (in Japanes). Clin Pharmacol Ther.

[CR21] Ogura Y, Gonsho A, Cyong JC, Orimo H (2004). Clinical trial of risedronate in Japanese volunteers: single and multiple oral dose studies. J Bone Miner Metab.

[CR22] Ohno K, Mori K, Orita M, Takeuchi M (2011). Computational insights into binding of bisphosphates to farnesyl pyrophosphate synthase. Curr Med Chem.

[CR23] Orriss IR, Key ML, Brandao-Burch A, Patel JJ, Burnstock G, Arnett TR (2012). The regulation of osteoblast function and bone mineralisation by extracellular nucleotides: The role of p2x receptors. Bone.

[CR24] Pellegatti P, Falzoni S, Donvito G, Lemaire I, Di Virgilio F (2011). P2X7 receptor drives osteoclast fusion by increasing the extracellular adenosine concentration. FASEB J.

[CR25] Reginster J, Minne HW, Sorensen OH, Hooper M, Roux C, Brandi ML, Lund B, Ethgen D, Pack S, Roumagnac I, Eastell R (2000). Randomized trial of the effects of risedronate on vertebral fractures in women with established postmenopausal osteoporosis. Vertebral Efficacy with Risedronate Therapy (VERT) Study Group. Osteoporos Int.

[CR26] Rogers MJ (2003). New insights into the molecular mechanisms of action of bisphosphonates. Curr Pharm Des.

[CR27] Russell RG, Watts NB, Ebetino FH, Rogers MJ (2008). Mechanisms of action of bisphosphonates: similarities and differences and their potential influence on clinical efficacy. Osteoporos Int.

[CR28] Sakai D, Tong HS, Minkin C (1995). Osteoclast molecular phenotyping by random cDNA sequencing. Bone.

[CR29] Sistermans EA, de Kok YJ, Peters W, Ginsel LA, Jap PH, Wieringa B (1995). Tissue- and cell-specific distribution of creatine kinase B: a new and highly specific monoclonal antibody for use in immunohistochemistry. Cell Tissue Res.

[CR30] Takami M, Suda K, Sahara T, Itoh K, Nagai K, Sasaki T, Udagawa N, Takahashi N (2003). Involvement of vacuolar H^+^ -ATPase in incorporation of risedronate into osteoclasts. Bone.

[CR31] Teitelbaum SL (2007). Osteoclasts: what do they do and how do they do it?. Am J Pathol.

[CR32] Tsubaki M, Itoh T, Satou T, Imano M, Komai M, Ogawa N, Mukai J, Nishida S (2013). Nitrogen-containing bisphosphonates induce apoptosis of hematopoietic tumor cells via inhibition of Ras signaling pathways and Bim-mediated activation of the intrinsic apoptotic pathway. Biochem Pharmacol.

[CR33] Waguespack SG, Hui SL, White KE, Buckwalter KA, Econs MJ (2002). Measurement of tartrate-resistant acid phosphatase and the brain isoenzyme of creatine kinase accurately diagnoses type II autosomal dominant osteopetrosis but does not identify gene carriers. J Clin Endocrinol Metab.

[CR34] Wallimann T, Wyss M, Brdiczka D, Nicolay K, Eppenberger HM (1992). Intracellular compartmentation, structure and function of creatine kinase isoenzymes in tissues with high and fluctuating energy demands: the 'phosphocreatine circuit' for cellular energy homeostasis. Biochem J.

[CR35] Weinstein RS, Roberson PK, Manolagas SC (2009). Giant osteoclast formation and long-term oral bisphosphonate therapy. N Engl J Med.

[CR36] Whyte MP, Chines A, Silva DP, Landt Y, Ladenson JH (1996). Creatine kinase brain isoenzyme (BB-CK) presence in serum distinguishes osteopetroses among the sclerosing bone disorders. J Bone Miner Res.

[CR37] Yoneyama T, Fowler HL, Pendleton JW, Sforza PP, Lui CY, Iranmanesh A, Gerard RD (1989). Elevated levels of creatine kinase BB isoenzyme in three patients with adult osteopetrosis. N Engl J Med.

