# Supplementary material for: Induction of creatine kinase release from cultured osteoclasts via the pharmacological action of aminobisphosphonates
Source: Springerplus. 2015 Feb 3;4:59. doi: 10.1186/s40064-015-0848-3 (PMC4315803; doi:10.1186/s40064-015-0848-3)
Supplement: Additional file 1: — Induction of Creatine Kinase Release from Cultured Osteoclasts via the Pharmacological Action of Aminobisphosphonates. [file 40064_2015_848_MOESM1_ESM.docx]

Additional file 1

**Induction of Creatine Kinase Release from Cultured Osteoclasts via the Pharmacological Action of Aminobisphosphonates**

Makoto Tanaka, Hiroshi Mori, Ryoji Kayasuga, Kazuhito Kawabata

Supplemental Fig. S1. Light micrographic analysis of cultured osteoclasts treated with minodronic acid. Cultured bone slices with cells were subjected to toluidine blue staining. (a, b) Osteoclasts in the control group had clear ruffled borders and deep resorption cavities on cortical bone slices, and contained many vesicles and vacuoles. (c, d) Osteoclasts treated with minodronic acid had no ruffled borders and had apoptotic condensed nuclei that were stained strongly by toluidine blue.


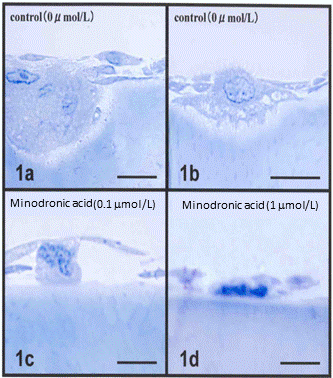


Supplemental Fig. S2. Cultured osteoclasts treated with minodronic acid. (a, b) Osteoclasts treated with 0.1 µmol/L minodronic acid had no ruffled borders, even for cells bound to the bone surface. Osteoclasts treated with 1 µmol/L minodronic acid did not make contact with bone.


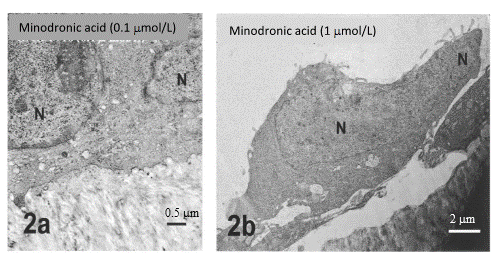


Materials and Methods

The pit assay was performed with the presence of minodronic acid in the culture medium. Cultured bone slices with cells were fixed in 2% paraformaldehyde and 2.5% glutaraldehyde containing 0.07 mmol/L phosphate buffer (pH 7.3) for 10 min, and washed with 0.1 mol/L phosphate buffer. The slices were decalcified in 5% EDTA at 4°C and embedded in EPON 812 using a routine procedure. Semi-ultrathin and ultrathin sections were prepared (Leica Ultra Cut, Leica Microsystems, Vienna, Austria). The semi-ultrathin sections were stained with toluidine blue and evaluated under light microscopy (Zeiss Axioplan 2, Zeiss, Jena, Germany). The ultrathin sections were evaluated using transmission electron microscopy (H-7600, Hitachi, Japan).
